# Bidirectional Relationship Between Osteoarthritis and Periodontitis: A Population-Based Cohort Study Over a 15-year Follow-Up

**DOI:** 10.3389/fimmu.2022.909783

**Published:** 2022-07-25

**Authors:** Kevin Sheng-Kai Ma, Jung-Nien Lai, Eshwar Thota, Hei-Tung Yip, Ning-Chien Chin, James Cheng-Chung Wei, Thomas E. Van Dyke

**Affiliations:** ^1^ Department of Epidemiology, Harvard T.H. Chan School of Public Health, Boston, MA, United States; ^2^ Center for Global Health, Perelman School of Medicine, University of Pennsylvania, Philadelphia, PA, United States; ^3^ Graduate Institute of Biomedical Electronics and Bioinformatics, National Taiwan University, Taipei, Taiwan; ^4^ School of Chinese Medicine, College of Chinese Medicine, China Medical University, Taichung, Taiwan; ^5^ Department of Chinese Medicine, China Medical University Hospital, Taichung, Taiwan; ^6^ Management office for Health Data, China Medical University Hospital, Taichung, Taiwan; ^7^ College of Medicine, China Medical University, Taichung, Taiwan; ^8^ Department of Orthopedics, Taichung Veterans General Hospital, Taichung, Taiwan; ^9^ Department of Orthopedics, Antai Tian-Sheng Memorial Hospital, Pingtung, Taiwan; ^10^ Division of Allergy, Immunology and Rheumatology, Chung Shan Medical University Hospital, Taichung, Taiwan; ^11^ Institute of Medicine, Chung Shan Medical University, Taichung, Taiwan; ^12^ Graduate Institute of Integrated Medicine, China Medical University, Taichung, Taiwan; ^13^ Center for Clinical and Translational Research, Forsyth Institute, Cambridge, MA, United States; ^14^ Department of Oral Medicine, Infection, and Immunity, Harvard School of Dental Medicine, Boston, MA, United States

**Keywords:** cohort study, periodontitis, osteoarthritis, total knee replacement, total hip replacement, inflammation

## Abstract

**Objective:**

To identify the relationship between osteoarthritis and periodontitis.

**Methods:**

144,788 periodontitis patients and 144,788 propensity score-matched controls without history of periodontitis were enrolled in this cohort study. A Cox proportional hazard model was used to estimate the risk of osteoarthritis. Survival analysis was utilized to assess the time-dependent effect of periodontitis on osteoarthritis. Age and gender were stratified to identify subgroups at risk. A symmetrical case-control analysis was designed to determine the relationship between present periodontitis and history of osteoarthritis.

**Results:**

Patients with periodontitis had higher risk of osteoarthritis (hazard ratio, HR =1.15, 95% CI =1.12–1.17, p < 0.001) and severe osteoarthritis that led to total knee replacement or total hip replacement (TKR/THR) (HR =1.12, 95% CI =1.03–1.21, p < 0.01) than controls, which was time-dependent (log-rank test p < 0.01). The effect of periodontitis on osteoarthritis was significant in both genders and age subgroups over 30 years-old (all p < 0.001). Among them, females (HR=1.27, 95% CI = 1.13–1.42, p < 0.001) and patients aged over 51 (HR= 1.21, 95% CI =1.10-1.33, p < 0.001) with periodontitis were predisposed to severe osteoarthritis. In addition, periodontitis patients were more likely to have a history of osteoarthritis (odds ratio = 1.11, 95% CI = 1.06 - 1.17, p < 0.001).

**Conclusions:**

These findings suggest an association between periodontitis and a higher risk of osteoarthritis, including severe osteoarthritis that led to TKR/THR. Likewise, periodontitis is more likely to develop following osteoarthritis. A bidirectional relationship between osteoarthritis and periodontitis was observed.

## Key Messages

1. Periodontitis was independently associated with a significantly higher risk of osteoarthritis than did controls.
2. Females and those aged over 50 with periodontitis were predisposed to severe osteoarthritis that needed surgery.
3. An independent bidirectional relationship between osteoarthritis and periodontitis was observed.

## Introduction

Osteoarthritis is characterized by progressive deterioration of articular cartilage with remodelling and proliferation of the bone beneath it (1–3), which serves as one of the leading causes of disability (1). Following the deterioration of articular cartilage, worsening osteoarthritis is one major indication for total joint replacement, with other indications including rheumatoid arthritis (RA) (2, 3). Among well-known etiological factors of osteoarthritis, primary osteoarthritis is caused by the malfunctioning synthetic function of hyaline cartilage, for which obesity and overloading contribute to its progression (1). Osteoarthritis can as well be secondary to causes such as instability of joint due to ligamentous laxity, neuropathies following diabetes mellitus (DM) (4, 5), inflammatory conditions following RA (6) or gout (3, 7), and aseptic necrosis (8). Mechanical insults in the form of trauma also fall into the secondary form of post-traumatic osteoarthritis, in which mechanical overload is then associated with a failure of the axis in the sense of valgosity and varosity of the knee joint (1). Apart from these recognized etiologies, it has been recently proposed that complement-mediated inflammatory cascades play a central role in osteoarthritis progression (9). That is, in addition to the conventional degenerative model (1), our knowledge of osteoarthritis pathogenesis has been expanded with an inflammation-dependent theory (9).

Periodontitis is an oral disease characterized by progressive inflammatory destruction of the periodontium and alveolar bone caused by biofilm-forming micro-organisms (10, 11). Such destructive inflammation is driven by complement-dependent mechanisms following oral microbial dysbiosis (12), and may translocate out of the oral cavity (10, 13–15). Clinical signs of periodontitis include gingival inflammation, alveolar bone loss, tooth mobility and eventually tooth loss (16). Periodontitis has been suggested to be a risk factor for systemic inflammatory conditions such as RA, even after considering known comorbidities (17); the underlying mechanism of which involve anti–citrullinated protein antibody (ACPA) formation by the periodontitis-associated pathogen *Porphyromonas gingivalis* (11). Likewise, periodontitis has been demonstrated to predispose to type 2 diabetes (14, 18–20), through mechanisms including metastatic inflammation in response to periodontal bacteria invasion (10, 18). These findings suggest a role for periodontal micro-organisms in systemic inflammation (17, 18) that may serve as the etiology of osteoarthritis acting through both complement cascades and periodontitis-associated systemic inflammation.

Failure of knee joint prosthesis may result from bacterial infection (2, 3), including oral bacteria that exist in the joint synovial samples from both natural and artificial joint tissues (13, 15). That is, there is an association between periodontitis and worse prognosis of total knee replacement (TKR) surgery (13, 15, 21, 22). Moreover, mechanical debridement to reduce oral bacteria has been proposed to reduce TKR failure (21, 22). This is consistent with our knowledge that periodontitis is associated with high risk of peri-prosthetic infection of alveolar bone implants, also known as peri-implantitis; as well as our knowledge of the proven efficacy of mechanical debridement for peri-implantitis management (23). Although the effect of periodontitis on risk of RA (24) and worse prognosis of TKR (13, 15, 21, 22) has been suggested, so far no studies have reported whether periodontitis as an chronic low-grade inflammatory event may be an independent risk factor of osteoarthritis in the general population. Given both the fact that bacterial invasion to the synovium could trigger complement cascades, and that periodontitis may exacerbate systemic inflammatory diseases that may drive osteoarthritis (11, 17, 25, 26), we conducted this population-based cohort study to identify whether periodontitis is a risk factor for osteoarthritis.

## Materials and Methods

### Study Design and Data Sources

This retrospective cohort study used data from the Longitudinal Health Insurance Database (LHID), a registry that includes all claimed diagnoses and treatments from outpatient visits, as well as emergency and hospitalization medical records. From LHID, one million randomly sampled eligible patients with periodontitis diagnosed between 1997-2013 were enrolled.

To select non-periodontitis controls without bias, propensity score matching was applied. The propensity score match using strata was performed by adjusting for age, gender, socioeconomic variables, including income, occupation, and healthcare accessibility in the residential area, underlying comorbidities, including systemic lupus erythematosus (SLE), RA, obesity, DM, hypertension, hyperlipidaemia, chronic liver disease, osteonecrosis, Paget’s disease, and hypothyroidism, and the year of periodontitis onset or matched-year for controls, to control for confounding factors between the periodontitis and non-periodontitis groups. The propensity score is a probability estimated through logistic regression (27–30), with which the cases and controls were matched on a 1:1 basis. To ensure the selection of the matched periodontitis and non-periodontitis cases was not biased, standard mean difference (SMD) was derived for the comparison between the cases and the controls. When the SMDs were equal to or less than 0.05, the characteristics of both groups were considered similar (25).

To assure the validity and consistency of the diagnosis used in the cohort, both osteoarthritis and periodontitis were confirmed in at least two outpatient visits or at least one inpatient discharge note within one year; all medical records, along with the diagnoses, were peer-reviewed for quality control by rheumatologists and dentists, respectively. Moreover, the validity of diagnoses has been confirmed in previous studies (31–33).The database was de-identified, and the current study was approved by the Institutional Review Board of Chung Shan Medical University Hospital (approval number CS15134).

### Case Definition for the Periodontitis Cohort

To determine whether periodontitis was associated with higher risks of osteoarthritis, medical records of patients diagnosed with periodontitis during 1997 to 2013 were retrieved from LHID. The occurrence of osteoarthritis after periodontitis onset was compared with that of propensity score-matched controls without periodontitis. The diagnosis of periodontitis was made following periodontal examinations that evaluated: (1) probing depth ≥ 5 mm in at least 4 teeth with each ≥ 1 site, (2) clinical attachment level (CAL) loss ≥5 mm at the same site, and (3) observed bleeding upon stimulus. The periodontal evaluations and diagnoses of periodontal diseases were made by dentists from medical centres, community hospitals and private dental clinics; the diagnosis of chronic periodontitis was confirmed in at least two outpatient visits within two years and peer-reviewed by other dentists to ensure its validity and consistency. The enrolled participants were followed up until the occurrence of osteoarthritis, December, 2013, or withdrawal, whichever occurred first.

To ensure each observed osteoarthritis case developed after periodontitis, we excluded the following patients: (1) patients with missing data throughout the period, including demographic information, comorbidity covariates, and death or loss to follow-up, (2) edentulous patients, (3) patients with tobacco addiction or alcoholism, (4) patients who throughout the study period had been diagnosed with osteoarthritis before periodontitis onset, (5) those whose diagnoses were made before 2000 or after 2012.

### Outcome Measurement for the Periodontitis Cohort

The diagnosis of osteoarthritis, which was the primary outcome of the periodontitis cohort, was made following the American College of Rheumatology (ACR) diagnostic criteria for osteoarthritis of the hand (26), hip (27), and knee (28, 29). A sensitivity analysis restricting the outcome of interest to only severe osteoarthritis that resulted in TKR or total hip replacement (THR) was conducted, for which osteoarthritis had to be the main diagnosis in the discharge notes of TKR/THR. Accordingly, the subsequent risks of receiving TKR/THR for osteoarthritis following periodontitis throughout the study period, as parameterized by adjusted hazard ratios (aHRs), was derived.

To identify the subgroups at risk of developing osteoarthritis and severe osteoarthritis following periodontitis, stratification of the analyses based on age and sex were carried out.

### The Osteoarthritis Case-Control Analysis and Follow-Up For Periodontitis Development

To identify whether the relationship between osteoarthritis and periodontitis was bidirectional or unidirectional, a symmetrical case-control analysis was designed parallel to the periodontitis cohort. Cases eligible for osteoarthritis case-control analysis were patients with periodontitis, for which their past history of osteoarthritis was retrieved from LHID. Propensity score-matched controls in the case-control analysis were selected based on demographic characteristics and comorbidities at baseline of periodontitis onset. Adjusted variables in propensity score matching were identical to those matched in the above-mentioned cohort design, and in the above-mentioned sensitivity analysis. The primary outcome, set as the history of osteoarthritis before periodontitis onset, was compared with that of propensity score-matched non-periodontitis controls, for which the odds ratios (ORs) were derived. Patients who had been diagnosed with periodontitis prior to osteoarthritis onset were excluded.

### Statistical Analysis

Baseline demographics and comorbidities at periodontitis onset were compared through chi-square tests for categorical variables and t-tests for continuous variables. Kaplan-Meier survival analysis (34) was used to compute the cumulative incidence of osteoarthritis, and a log-rank test was used to test the significance of differences between cases of periodontitis and propensity score-matched controls. Cox proportional hazard regression with time-dependent periodontitis and comorbidity information using the counting process was used to produce the HRs of osteoarthritis between periodontitis cases and propensity score-matched non-periodontitis controls. All analyses were conducted using SPSS version 18.0 (SPSS Inc., Chicago, IL, USA).

## Results

### Basic Demographics of the Study Subjects

**Figure 1:**
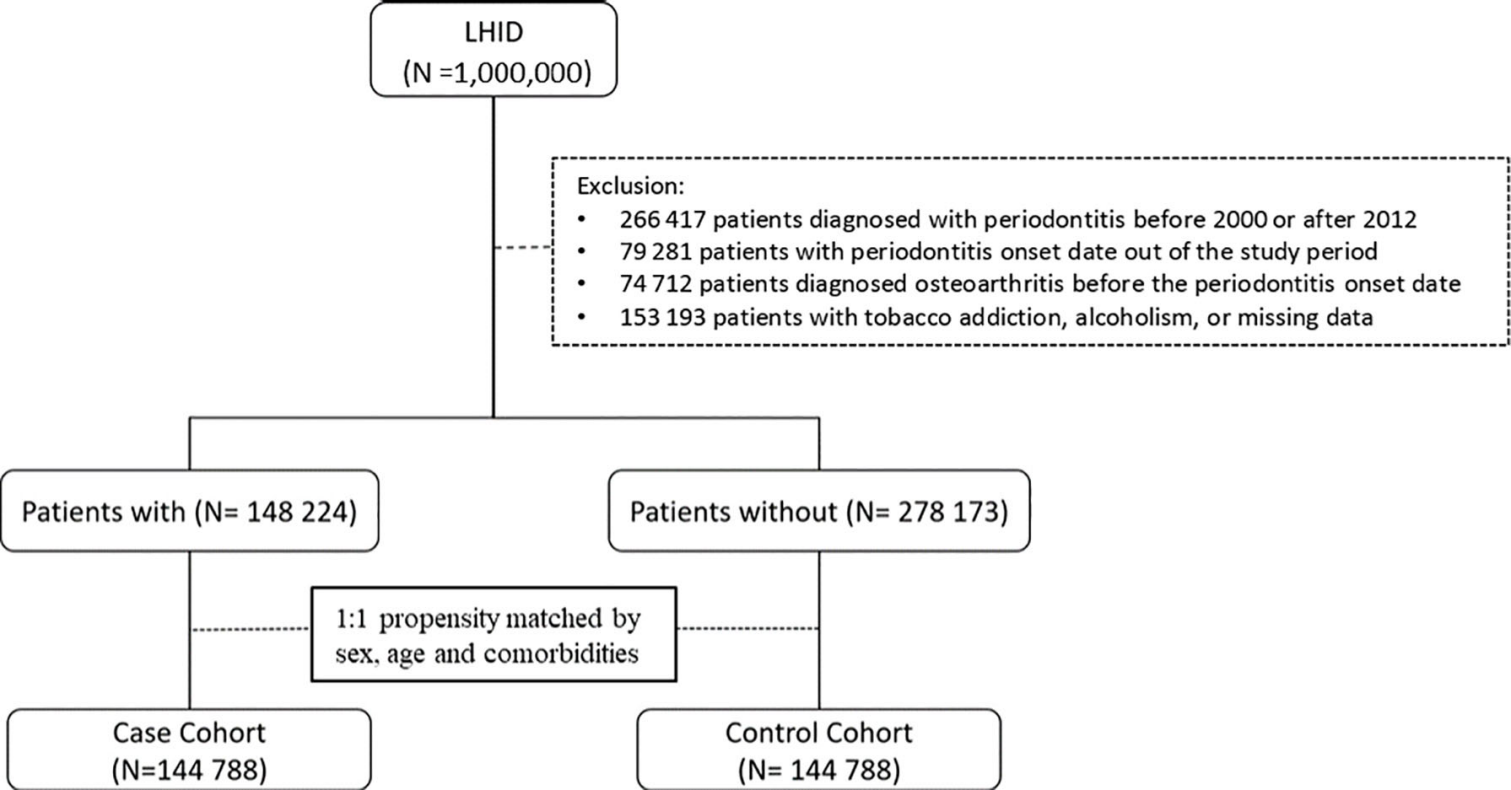
Patient selection flowchart for the periodontitis cohort.

For the periodontitis cohort, a total of 148,224 eligible patients who were newly diagnosed with periodontitis were identified from LHID (**Figure 1**). After propensity score matching, 144,788 subjects from periodontitis and the non-periodontitis group were selected (**Figure 1**) with a matched mean age of 39 ± 15 years for the final cohort (**Table 1**). There was no statistically significant difference for demographic variables and incidence of comorbidities between the periodontitis group and the non-periodontitis group (all SMDs>0.05) (**Table 1**).

**Table 1:** Baseline characteristics for the periodontitis and the non-periodontitis cohorts after propensity score matching.

Variables	Periodontitis					SMD
	No (N = 144 788)			Yes (N = 144 788)		
	n	%		n	%	
Gender						0.01
Female	66756	46%		67304	46%	
Male	78032	54%		77484	54%	
Age, year						
18-30	51354	35%		50052	35%	0.02
31-40	31955	22%		31145	22%	0.01
41-50	29552	20%		31247	22%	0.03
>=51	31927	22%		32344	22%	0.01
mean, (SD)*	38.6	(15.0)		38.7	(14.8)	0.01
Comorbidities						
obesity	100	0.07%		155	0.11%	0.01
SLE	7	0.00%		12	0.01%	0.004
Septic arthritis	0	0%		0	0%	
Rheumatoid arthritis	739	1%		921	1%	0.02
Hypertension	19153	13%		18944	13%	0.004
diabetes mellitus	8926	6%		9090	6%	0.01
Hyperlipidemia	11026	8%		10888	8%	0.004
CLD	19979	14%		20125	14%	0.003
Osteonecrosis	3	0.002%		5	0.003%	0.003
Paget’s disease of bone	9	0.01%		10	0.01%	0.001
Underactive thyroid	206	0.14%		300	0.21%	0.02

*, examined by Student’s t-test; SMD, standard mean difference; SMDs ≤ 0.05 were considered similar. SLE, systemic lupus erythematosus; CLD, chronic liver disease.

### Risk of Osteoarthritis in Patients With Periodontitis

**Figure 2:**
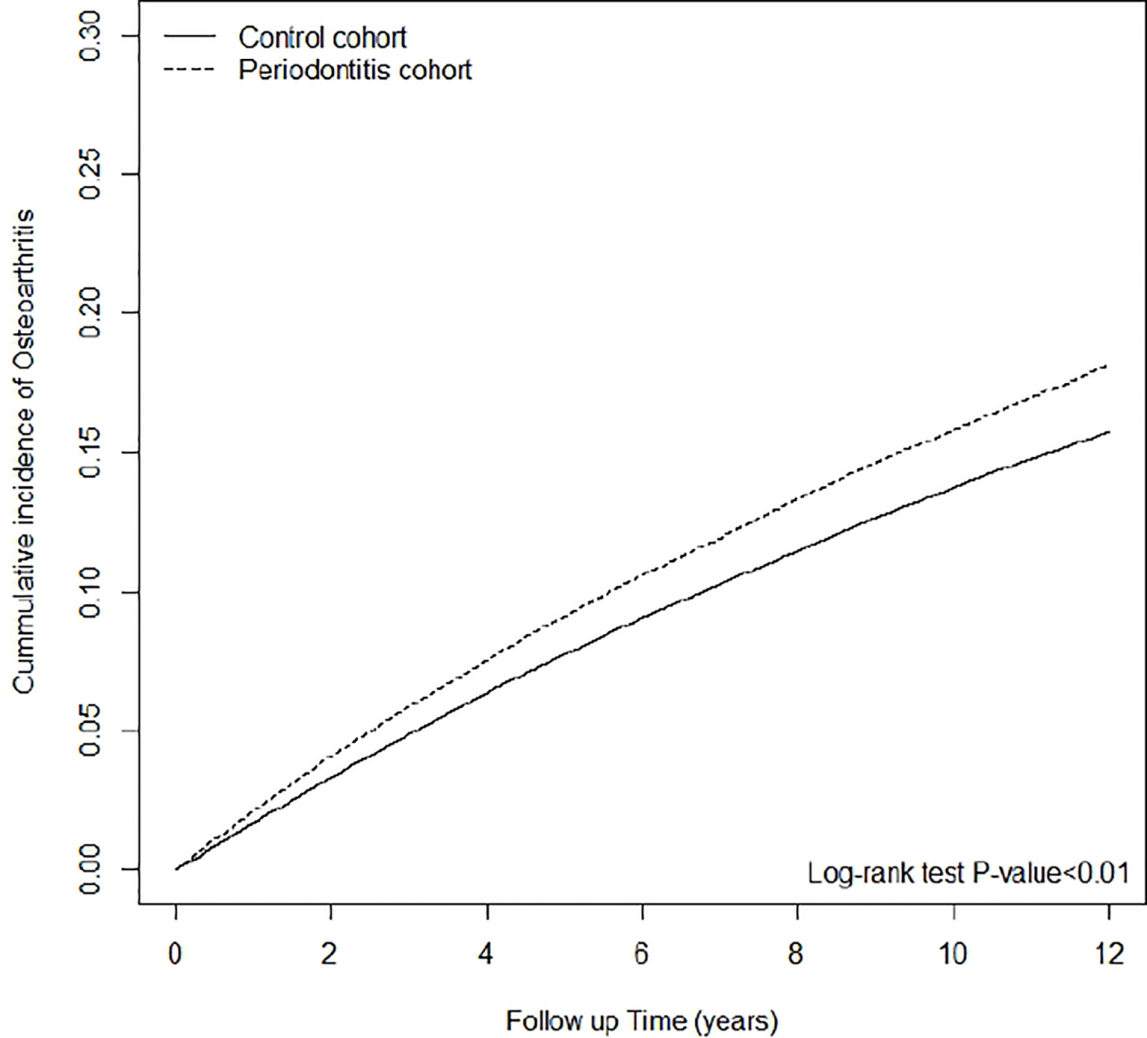
Cumulative incidence of osteoarthritis in patients with and without periodontitis.

Among the 144,788 periodontitis cases enrolled in this study, 21,021 cases developed osteoarthritis after the onset of periodontitis, which was identified over 1,218,644 observed person-years. The incidence rate (IR) of osteoarthritis in patients who had periodontitis was significantly higher than the non-periodontitis cohort (1.72 v.s. 1.48 per 1,000 person-years) (p <0.001). Patients who had periodontitis had a significantly higher risk of osteoarthritis when compared to the non-periodontitis controls (adjusted HR=1.15, 95%CI=1.12-1.17) (**Table 2**). To sum up, the cumulative incidence of osteoarthritis in the periodontitis group was significantly higher than that of the non-periodontitis group, which was statistically significant (log-rank test p < 0.01) (**Figure 2**).

**Table 2:** Multiple Cox proportional model for risks of osteoarthritis (OA) in the periodontitis cohort.

	OA						OA with TKR/THR			
Variables	n	PY	IR	aHR^†^	(95% CI)	n	PY	IR	aHR^†^	(95% CI)
Non-periodontitis	17598	1191717	1.48	1.00	–	1069	1192884	0.90	1.00	–
Periodontitis	21021	1218644	1.72	1.15	(1.12,1.17)***	1199	1219829	0.98	1.12	(1.03,1.21)**
Gender										
Female	21169	1111895	1.90	1.00	–	1264	1112910	1.14	1.00	–
Male	17450	1298465	1.34	0.66	(0.65,0.67)***	1004	1299803	0.77	0.58	(0.53,0.62)***
Age, year										
18-30	3411	904164	0.38	1.00	–	71	904303	0.08	1.00	–
31-40	5253	575982	0.91	2.32	(2.23,2.43)***	189	576353	0.33	4.18	(3.18,5.49)***
41-50	10342	506429	2.04	4.84	(4.66,5.03)***	376	506934	0.74	8.89	(6.90,11.5)***
>=51	19613	423785	4.63	9.59	(9.23,9.97)***	1632	425122	3.84	39.6	(31.1,50.5)***
Comorbidities										
Obesity										
No	38587	2409187	1.60	1.00	–	2268	2411539	0.94		
Yes	32	1174	2.73	1.18	(0.83,1.66)	0	1174	0.00		
SLE										
No	38617	2410215	1.60			2268	2412568	0.94		
Yes	2	145	1.38			0	145	0.00		
Rheumatoid arthritis										
No	38112	2399522	1.59	1.00	–	2209	2401773	0.92	1.00	–
Yes	507	10838	4.68	1.56	(1.43,1.71)***	59	10940	5.39	2.69	(2.07,3.48)***
Hypertension										
No	26935	2164469	1.24	1.00	–	1283	2166031	0.59	1.00	–
Yes	11684	245892	4.75	1.46	(1.43,1.50)***	985	246682	3.99	1.97	(1.79,2.16)***
Diabetes mellitus										
No	33361	2295345	1.45	1.00	–	1868	2297335	0.81	1.00	–
Yes	5258	115015	4.57	1.09	(1.06,1.13)***	400	115378	3.47	1.05	(0.93,1.18)
Hyperlipidemia										
No	32699	2271556	1.44	1.00	–	1832	2273566	0.81	1.00	–
Yes	5920	138804	4.26	1.10	(1.06,1.13)***	436	139147	3.13	1.00	(0.89,1.13)
CLD										
No	29683	2113086	1.40	1.00	–	1709	2114974	0.81	1.00	–
Yes	8936	297275	3.01	1.39	(1.36,1.43)***	559	297739	1.88	1.32	(1.19,1.46)***
Osteonecrosis										
No	38615	2410325	1.60	1.00	–	2265	2412677	0.94	1.00	–
Yes	4	35	11.3	4.43	(1.66,11.81)**	3	35	84.8	39.1	(12.6,122)***
Paget’s disease of bone										
No	38613	2410246	1.60	1.00	–	2267	2412599	0.94	1.00	–
Yes	6	114	5.27	1.63	(0.73,3.62)	1	114	8.78	3.01	(0.43,21.3)
Underactive thyroid										
No	38519	2407116	1.60	1.00	–	2266	2409468	0.94		
Yes	100	3245	3.08	1.07	(0.88,1.30)	2	3245	0.62		

n, number of events; PY, person-years; IR, incidence rate per 1,000 person-years; cHR, crude hazard ratio; aHR, adjusted hazard ratio; SLE, systemic lupus erythematosus; CLD, chronic liver disease; TKR, total knee replacement; THR, total hip replacement.

*: p-value <0.05; **: p-value <0.01; ***: p-value <0.001; †: adjusted by gender, age, and all comorbidities.

### Subgroup Analysis for the Risk of Osteoarthritis in Patients with Periodontitis, Stratified by Age and Gender

Findings of the subgroup analysis suggested that the association between periodontitis and the risk of developing osteoarthritis was significant for both genders. Among them, the risk of osteoarthritis for 9,626 male periodontitis patients (aHR=1.18, 95% CI=1.15-1.22, p < 0.001) was higher than that for 11,395 female periodontitis patients (aHR=1.11, 95% CI = 1.09-1.15, p < 0.001) (**Table 3**).

**Table 3:** Subgroup analyses on risks of osteoarthritis following periodontitis, based on sex and age stratification.

	Non-periodontitis				Periodontitis						
Variables	n	PY	IR		n	PY	IR	cHR	(95% CI)	aHR^†^	(95% CI)
Gender											
Female	9774	549242	1.78		11395	562654	2.03	1.14	(1.11,1.17)***	1.11	(1.09,1.15)***
Male	7824	642475	1.22		9626	655990	1.47	1.21	(1.17,1.24)***	1.18	(1.15,1.22)***
Age, year											
18-30	1659	453385	0.37		1752	450779	0.39	1.06	(0.99,1.14)	1.07	(1,1.14)
31-40	2450	288011	0.85		2803	287971	0.97	1.14	(1.08,1.21)***	1.14	(1.08,1.21)***
41-50	4576	243394	1.88		5766	263035	2.19	1.17	(1.12,1.21)***	1.17	(1.12,1.21)***
>=51	8913	206927	4.31		10700	216859	4.93	1.15	(1.12,1.18)***	1.15	(1.12,1.18)***

n, number of events; PY, person-years; IR, incidence rate per 1,000 person-years; cHR, crude hazard ratio; aHR, adjusted hazard ratio.

*: p-value <0.05; **: p-value <0.01; ***: p-value <0.001.

†: adjusted by gender, age, and comorbidities.

Likewise, subgroup analysis based on age stratification showed that the risk of osteoarthritis in the periodontitis group was particularly high among patients aged over 30. Specifically, periodontitis patients aged between 31-40 (n=2,803, aHR=1.14, 95% CI = 1.08-1.21, p < 0.001), aged between 41-50 (n=5,766, aHR=1.17, 95% CI=1.12-1.21, p < 0.001), and aged over 51 (n=10,700, aHR = 1.15, 95% CI = 1.12-1.18, p < 0.001) had significantly higher risk of developing osteoarthritis. In comparison, the association between periodontitis and risk of osteoarthritis in those aged 18-30 (n = 1,752, aHR = 1.07, 95% CI = 1-1.14) was not significant (**Table 3**).

### Sensitivity Analysis on the Risk of TKR/THR for Severe Osteoarthritis Following Periodontitis

Among the 21,021 cases that developed osteoarthritis following periodontitis onset, 1,199 patients received TKR/THR. The risk of osteoarthritis-associated TKR/THR in periodontitis patients was significantly higher than the non-periodontitis controls (aHR = 1.12, 95% CI = 1.03-1.21, p < 0.01) (**Table 2**). Likewise, the incidence rate (IR) of severe osteoarthritis that needed TKR/THR in patients who had periodontitis was significantly higher than the non-periodontitis controls (0.98 v.s. 0.90 per 1,000 person years) (p <0.01) (**Table 2**).

The subgroup analysis suggested that the association between periodontitis and the risk of severe osteoarthritis that needed TKR/THR was particularly significant for females (aHR = 1.27; 95% CI = 1.13 – 1.42) (**Table 4**). Likewise, subgroup analysis based on age stratification showed that the risk of osteoarthritis in the periodontitis group was significantly higher among patients aged over 51 (n = 878, aHR = 1.21, 95% CI = 1.10-1.33, p < 0.001). In comparison, the association between periodontitis and risks of osteoarthritis for those aged below 50 and males was not significant (**Table 4**).

**Table 4:** Subgroup analyses on risks of severe osteoarthritis that led to total knee or total hip replacement following periodontitis, based on sex and age stratification.

	Non-periodontitis				Periodontitis						
Variables	n	PY	IR		n	PY	IR	cHR	(95% CI)	aHR^†^	(95% CI)
Gender											
Female	577	549721	1.05		687	563189	1.22	1.19	(1.06, 1.32)**	1.27	(1.13, 1.42)***
Male	492	643163	0.76		512	656640	0.78	1.04	(0.92, 1.18)	1.05	(0.93, 1.19)
Age, year											
18-30	36	453458	0.08		35	450845	0.08	0.98	(0.62, 1.56)	0.96	(0.60, 1.54)
31-40	94	288208	0.33		95	288146	0.33	1.02	(0.77, 1.36)	0.99	(0.75, 1.32)
41-50	185	243654	0.76		191	263280	0.73	0.98	(0.80, 1.20)	0.98	(0.80, 1.20)
>=51	754	207564	3.63		878	217558	4.04	1.17	(1.06, 1.29)**	1.21	(1.10, 1.33)***

n, number of events; PY, person-years; IR, incidence rate per 1,000 person-years; cHR, crude hazard ratio; aHR, adjusted hazard ratio.

*: p-value <0.05; **: p-value <0.01; ***: p-value <0.001.

†: adjusted by gender, age, rheumatoid arthritis, hypertension, diabetes mellitus, hyperlipidemia, CLD, osteonecrosis and Paget’s disease of bone.

### Current Periodontitis and the History of Osteoarthritis

Patients with periodontitis were more likely to have a history of osteoarthritis (adjusted OR, aOR = 1.11, 95% CI = 1.06 - 1.17, p < 0.001), compared to non-periodontitis controls. This was identified in the symmetrical case-control analysis consisting of 51,551 periodontitis patients (**Supplementary Table S1**), in which 4,783 cases (9.3%) had osteoarthritis before periodontitis onset. The OR of osteoarthritis history was significantly higher among periodontitis patients than that of non-periodontitis controls (9.3% v.s. 8.0%) (**Table 5**).

**Table 5:** Odds ratios of osteoarthritis developed within 5 year before periodontitis.

	Osteoarthritis						
Variables	N	n	%	cOR	(95% CI)	aOR	(95% CI)
Non-periodontitis	51551	4124	8.0%	1.00	–	1.00	–
Periodontitis	51551	4783	9.3%	1.18	(1.13,1.23)***	1.11	(1.06,1.17)***
Gender							
Female	48229	5425	11%	1.00	–	1.00	–
Male	54873	3482	6%	0.53	(0.51,0.56)***	0.56	(0.53,0.59)***
Age, year							
18-30	33421	330	1.0%	1.00	–	1.00	–
31-40	16974	396	2.3%	2.40	(2.07,2.78)***	2.11	(1.82,2.45)***
41-50	19738	979	5.0%	5.23	(4.61,5.94)***	3.59	(3.16,4.08)***
≥51	32969	7202	21.8%	28.0	(25.1,31.3)***	12.2	(10.9,13.7)***
Comorbidities							
Obesity							
No	102742	8845	9%	1.00	–	1.00	–
Yes	360	62	17%	2.21	(1.68,2.91)***	1.07	(0.78,1.47)
SLE							
No	103081	8904	9%	1.00	–		
Yes	21	3	14%	1.78	(0.53,6.01)		
Rheumatoid arthritis							
No	101430	8180	8%	1.00	–	1.00	–
Yes	1672	727	43%	8.77	(7.94,9.69)***	3.69	(3.3,4.14)***
Hypertension							
No	83457	3643	4%	1.00	–	1.00	–
Yes	19645	5264	27%	8.02	(7.66,8.4)***	2.12	(2,2.24)***
Diabetes mellitus							
No	92999	6046	7%	1.00	–	1.00	–
Yes	10103	2861	28%	5.68	(5.4,5.98)***	1.25	(1.17,1.33)***
Hyperlipidemia							
No	89035	5045	6%	1.00	–	1.00	–
Yes	14067	3862	27%	6.30	(6.01,6.6)***	1.64	(1.55,1.74)***
CLD							
No	85613	5398	6%	1.00	–	1.00	–
Yes	17489	3509	20%	3.73	(3.56,3.91)***	1.64	(1.55,1.73)***
Osteonecrosis							
No	103090	8901	9%	1.00	–	1.00	–
Yes	12	6	50%	10.5	(3.41,32.82)***	7.65	(2.07,28.2)**
Paget’s disease of bone							
No	103087	8902	9%	1.00	–	1.00	–
Yes	15	5	33%	5.29	(1.81,15.48)**	2.80	(0.85,9.21)
Underactive thyroid							
No	102646	8804	9%	1.00	–	1.00	–
Yes	456	103	23%	3.11	(2.5,3.88)***	1.23	(0.96,1.58)

N, number of participants; n, number of events; cOR, crude odds ratio; aOR, adjusted odds ratio; SLE, systemic lupus erythematosus; CLD, chronic liver disease. *: p-value < 0.05; **: p-value < 0.01; ***: p-value < 0.001.

Among all enrolled patients, 330 patients (1.0%) below the age of 30 had osteoarthritis before periodontitis onset. Compared with those aged below 30, patients with periodontitis aged between 31-40 (n=396, aOR=2.11, 95% CI = 1.82-2.45), patients aged between 41-50 (n=979, aOR=3.59, 95% CI = 3.16-4.08), and aged over 51 (n=7,202, aOR=12.2, 95% CI = 10.9-13.7) were associated with higher incidence of osteoarthritis history (**Table 5**). Moreover, females were associated with higher incidence of osteoarthritis history (n=5,425, 11%), compared to males (n=3,482, aOR= 0.56, 95% CI =0.53-0.59) (**Table 5**). Both the age and sex distribution of osteoarthritis were in line with previous studies (1, 4).

## Discussion

The findings of this cohort study suggest that periodontitis and osteoarthritis are linked in a bidirectional pattern. After propensity score matching for potential confounding factors including common risk factors for osteoarthritis, the Cox proportional hazard model revealed that the risk of osteoarthritis was significantly higher for patients who had periodontitis than that for the non-periodontitis controls. These findings were time-dependent, as suggested by the log-rank test of the Kaplan–Meier curve, and persisted in the sensitivity analysis, in which the criteria of osteoarthritis was confined to surgically treated osteoarthritis with TKR/THR. Patients of both genders and all age groups aged over 30 had higher risks for osteoarthritis following periodontitis; among them, women and those aged over 51 had higher risks for severe osteoarthritis that led to TKR/THR. In line with the epidemiology of osteoarthritis (1–4), the incidence rates of osteoarthritis were higher among females and the elders in our study. To the contrary, the association between periodontitis and the risk for osteoarthritis in males was higher following periodontitis. This is interesting as periodontitis is also more frequent and severe in males as opposed to females (35, 36). In the symmetrical case-control analysis, where the incidences of osteoarthritis history among periodontitis versus non-periodontitis participants were compared, periodontitis patients were more likely to have a history of osteoarthritis. Overall, increased risks of osteoarthritis and periodontitis were observed for patients with either disease.

Based on its etiologies, osteoarthritis could be classified into primary and secondary osteoarthritis. Primary osteoarthritis, caused by a disorder in the synthetic function of hyaline cartilage, comprises most of the cases. Although conventionally considered as a non-inflammatory arthritis (1), osteoarthritis has lately been suggested to be driven by complement-mediated inflammatory processes (9, 37) and has been managed as an inflammatory disease with medications including nonsteroidal antiinflammatory drugs (NSAIDs) (38). Hence, a paradigm shift of osteoarthritis pathogenesis has occurred towards an inflammatory-associated progression, which expands our understanding of the wear-and-tear theory (1). At the same time, infections originating in the oral cavity, with pathogens being isolated from the synovial fluid of patients operated for peri-prosthetic joint infection (PJI), have been suggested to contribute to a worse prognosis for TKR/THR (13, 22). These concepts (13, 15) have been applied to promote prophylactic management of periodontal diseases prior to joint replacement surgeries (21, 22). Given the fact that periodontopathic bacteria have been identified in joint synovium (10, 13–15), and that inflammatory complement cascades may be initiated by bacterial infection (39–41), it is possible that periodontitis could lead to or exacerbate the progression of osteoarthritis. In this first hypothesis, instead of obvious infection and inflammation that develops into septic arthritis (42), periodontal pathogens may cause or aggravate the inflammatory process in the joints, finally developing into osteoarthritis, or even severe osteoarthritis that requires surgical treatment.

Apart from being involved in idiopathic or primary osteoarthritis, periodontitis and periodontopathic bacterial invasion (43) have been shown to increase the risk of RA (7, 11, 17) and DM (5, 10, 14, 18, 20, 44, 45). These clinically evident correlations provide a plausible causal interpretation that may connect periodontitis to secondary osteoarthritis. Periodontal pathogens may trigger osteoarthritis indirectly through mechanisms including autoantibodies such as ACPA (11, 46), or humoral immunity (47–50) that involves ANCA and B cell activation upon bacterial infection (25).

Osteoarthritis could develop following periodontal pathogen invasion, which may further indicate an infection-associated model for osteoarthritis progression. The underlying mechanism of this relationship could be similar to odontogenic infections that lead to metastatic infection, metastatic inflammation, or endotoxin-driven metastatic injury (10, 14, 51). In this sense, our findings could be interpreted that periodontitis is an etiologic factor for osteoarthritis, which may trigger joint degeneration once metastatic injury is initiated. To further explore this potential relationship, intervention studies are required to determine whether mechanical debridement and surgical management for periodontal diseases leads to the primary prevention of osteoarthritis, the clinical improvement of osteoarthritis, the prevention of PJI in patients who had received TKR/THR.

Periodontitis has been suggested to be a comorbidity with knee osteoarthritis in a cross-sectional study (52). Although the underlying mechanisms are yet to be studied, potential common denominators might include underlying inflammatory traits that made those individuals susceptible to both osteoarthritis and periodontitis, with the two diseases being regarded as inflammatory diseases. Our findings may be interpreted as periodontitis serving as an early sign, or a predictor of osteoarthritis, which may be clinically identified prior to osteoarthritis onset.

Findings in the present study supported that osteoarthritis also increased the risk for future periodontitis. Whether this is a direct common susceptibility of related to impaired motor function is unknown. Osteoarthritis of the hands, knees, and hip has been suggested to result in impaired functional ability (40) that may result in poor oral hygiene due to a lack of daily activity and patient education (53), with excess biofilm formation on teeth and the periodontium, followed by increased risks of dental caries (54, 55) and periodontal diseases. This may explain our findings in the case-control analysis of the correlation between present periodontitis and a history of osteoarthritis. Prospective studies including more parameters for osteoarthritis measurement, such as the Kellgren-Lawrence (K&L) grading scale (56), is warranted, to precisely depict the observed association between osteoarthritis and periodontitis.

Some limitations of this study include the lack of periodontal charting data, including probing depth measurments, clinical attachment level measurments, or relevant local inflammatory biomarker measurments. These parameters may be of interest as they represent the degree of periodontal destruction underlying periodontitis (57), through which their simultaneous correlation with K&L scores would be reflective of the above-propsed reciprocal or bidirectional model. Additionally, even though we have observed the temporal association between periodontitis and risk of osteoarthritis through time-dependent survival analysis, we cannot infer causation as bacterial cultures based on synovial samples and inflammatory biomarkers in pateints with osteoarthritis were not available in our database. Accordingly, we advocate studies with both K&L scores and synovial samples from osteoarthritis-involved joints to validate our findings. Despite these potential limitations, in relation to the previous studies, which were mainly cross-sectional, our major strengths include a fairly large sample size in a real-world setting (58, 59). Also, propensity score matching was adopted in this longitudinal study to minimize the effect of potential confounding factors (60) on associations.

To our knowledge, this is the first study suggesting a long-term association between periodontitis and osteoarthritis. After adjusting for known comorbidities and covariates, the association between periodontitis and risk of osteoarthritis was significant, for both sexes, and all periodontitis patients aged over 30 years. Females with periodontitis and those aged over 50 with periodontitis were predisposed to severe osteoarthritis that led to TKR/THR. We advocate for more research on infection-associated osteoarthritis pathogenesis and clinical studies.

## Data Availability Statement

The raw data supporting the conclusions of this article will be made available by the authors, without undue reservation.

## Ethics Statement

The studies involving human participants were reviewed and approved by The institutional review board, chung shan medical university hospital. The ethics committee waived the requirement of written informed consent for participation.

## Author Contributions

KM , JW, and TD conceived and designed the study. KM and H-TY contributed to data analysis and interpretation. KM, J-NL, ET, and N-CC wrote the manuscript. All authors approved the final version of the manuscript to be published.

## Funding

This work was supported by a research grant from International Team for Implantology (fund no. 1577_2021 to KSM).

## Conflict of Interest

The authors declare that the research was conducted in the absence of any commercial or financial relationships that could be construed as a potential conflict of interest.

## Publisher’s Note

All claims expressed in this article are solely those of the authors and do not necessarily represent those of their affiliated organizations, or those of the publisher, the editors and the reviewers. Any product that may be evaluated in this article, or claim that may be made by its manufacturer, is not guaranteed or endorsed by the publisher.
