# Supplementary material for: Bidirectional Relationship Between Osteoarthritis and Periodontitis: A Population-Based Cohort Study Over a 15-year Follow-Up
Source: Front Immunol. 2022 Jul 25;13:909783. doi: 10.3389/fimmu.2022.909783 (PMC9358960; doi:10.3389/fimmu.2022.909783)
Supplement: Supplementary file 1 [file Table_1.docx]

**Supplementary Table S1.** Baseline characteristic of patients with and without periodontitis.

|  | Periodontitis | | | | |  |
| --- | --- | --- | --- | --- | --- | --- |
|  | No (N=51 551) | |  | Yes (N=51 551) | |  |
| Variables | n | % |  | n | % | p-value |
| Gender |  |  |  |  |  | 1.00 |
| Female | 24114 | 46.8 |  | 24115 | 46.8 |  |
| Male | 27437 | 53.2 |  | 27436 | 53.2 |  |
| Age, year |  |  |  |  |  | 1.00 |
| 18-30 | 16708 | 32.4 |  | 16713 | 32.4 |  |
| 31-40 | 8485 | 16.5 |  | 8489 | 16.5 |  |
| 41-50 | 9868 | 19.1 |  | 9870 | 19.1 |  |
| ≥51 | 16490 | 32.0 |  | 16479 | 32.0 |  |
| mean, (SD) | 41.76 | 17.1 |  | 41.7 | 17.1 | 1.00 |

matched by gender, age and index y
